# Reference values of gait using APDM movement monitoring inertial sensor system

**DOI:** 10.1098/rsos.170818

**Published:** 2018-01-10

**Authors:** Xin Fang, Chuandao Liu, Zhongli Jiang

**Affiliations:** 1 Department of Rehabilitation Medicine, The First Affiliated Hospital of Nanjing Medical University, Nanjing 210029, People's Republic of China; 2 Department of Rehabilitation Medicine, the First Affiliated Hospital of Soochow University, Suzhou 215006, People's Republic of China

**Keywords:** gait, reference values, inertial sensor, rehabilitation, initial contact angle, toe-off angle

## Abstract

Normal gait data reported show variability depending on specific equipment and techniques. Reference values of initial contact angle (ICA) and toe-off angle (TOA) are still lacking. We present a normative gait database of 292 healthy adults using the APDM Movement Monitoring inertial sensor system across a large age span of adulthood. Data were collected as participants completed a walk test for 2 min. Normalization was conducted and two factors were extracted by a factor analysis. Six reference gait variables under each factor were presented and the impacts of age, gender and BMI were evaluated by MANOVA and ANCOVA. ICA and TOA were highly correlated with speed and stride length. ICA was significantly larger in men, whereas larger TOA could be observed in women in all age groups but could not achieve significant difference. Overweight and obese adults walked at significantly lower speed, shorter stride length, reduced cadence and longer gait cycle duration. TOA was smaller in the obese group. However, the differences in ICA were not significant. Reference gait values described herein were valuable for identifying and interpreting gait phenomena using APDM®, contributing to rehabilitation of gait dysfunction.

## 1. Introduction

Walking is a highly complex procedure under the regulation and coordination of neural and musculoskeletal systems. Human gait is characterized by smooth and economical periodic movements [1]. Commonly used movement assessments in clinical and research work include subjective evaluations including observation and assessment scales and objective evaluations involving stopwatch, electronic goniometer, electronic walkway, force plate and optical three-dimensional motion analysis systems, with advantages accompanied by limitations [2]. Observation of gait relies highly on personal experience and is not easy to quantify. Ceiling effect and clinician bias exist in some rating scales. Although an optical three-dimensional motion analysis system can provide objective measurement of gait, it is much more expensive, complicated to use and time-consuming. It requires skilled technicians for laboratory use and is not fit for the clinical environment. An electronic walkway of short distance used in gait assessment is distinct from the daily living environment making it difficult to study gait symmetry and variability, which essential to study of gait dysfunction.

In recent years, a series of inertial sensor based gait analysis systems have been playing a growing role in human motion analysis [2]. A newly emerging one is the APDM Movement Monitoring inertial sensor system (Opals and Mobility Lab) [3]. Its portable body-worn monitoring sensors are characteristically of small size, low weight, good sensitivity, lower cost and easy to use. As long as a normative walkway is provided, it can objectively record and automatically analyse gait information from the tri-axial accelerometer, tri-axial gyroscope and magnetometer signals, applicable in the laboratory, clinic and, further, in community and family settings for continuous monitoring [4,5].

Basic gait parameters including cadence, walking speed and stride length were previously the most commonly studied by investigators. However, normal reference data of these parameters showed much variability [6–8], mainly caused by different equipment and techniques. To date, reference values for additional gait measures such as initial contact angle (ICA) and toe-off angle (TOA) are still lacking and worthy of investigation.

It is known that a full gait cycle starts from heel strike (sometimes referred to as initial contact) of one leg to subsequent ipsilateral heel strike. Toe-off marks the transition from stance to swing phase. ICA is the angle between the foot and the ground when left/right foot initially hits the ground. TOA is the angle between the foot and the ground near terminal stance and immediately before the foot leaves the ground. Published studies using inertial sensors mostly focused on producing approaches for accurate detection of gait events of initial contact and toe-off in walking and running over ground and on a treadmill [9–12]. Investigations using electronic walkway, force plate and other motion detection systems mainly addressed the muscle forces, joint loads and moment during initial contact and toe-off for evaluation and treatment of pathologies. For patients with stroke [13–15], impaired ankle power generation in late stance caused lower peak knee flexion in swing, thus lessened walking ability and raised tripping risk. With respect to patients with cerebral palsy [16], reduced rapid force generation of plantarflexion and dorsiflexion contributed to impaired gait function. Measurement of ICA and TOA can well reflect the plantarflexion and dorsiflexion movement of the ankle in walking, and may make valuable implications for fall after tripping, identification of pathological gait, assessment of rehabilitation intervention, and designs of prostheses and orthoses to restore locomotion functions of the disabilities. In addition, Holbein-Jenny *et al*. [17] studied six young adults and found increased cadences from 70 to 100 steps min^−1^ were associated with increased angle at heel strike. But only two of the six subjects experienced a significant effect of carrying load on foot contact angle. These results can serve to help develop the slip resistance measurements in order to reduce occupational and other slip and fall injuries. Indeed, it is important to build a reference database of ICA and TOA in healthy adults for better understanding and interpretation of gait phenomena. However, to date, very little has been published, especially a database using the inertial sensor system.

Thus, the purposes of this study were to present reference group data of 292 healthy participants, 20–89 years of age, in a 2-min walk test (2MWT) at natural speed by the APDM Movement Monitoring inertial sensor system as well as to identify how subject characteristics related to gait parameters. Reference group data are needed for clinicians and researchers to evaluate and interpret gait impairments, so as to draw up rehabilitation plans and training interventions.

## 2. Methods

### 2.1. Participants

Three hundred and twenty-one healthy subjects were recruited from university, companies, residential communities, retirement communities as well as medical staff and carers from inpatient rehabilitation settings. Exclusion criteria were: (i) cardiovascular or pulmonary pathologies; (ii) musculoskeletal or neurological diseases; (iii) musculoskeletal injuries within the last year; (iv) vestibular problems; (v) any other conditions affecting gait. Twenty-one were excluded based on medical history (myocardial infarction: one; pacemaker: one; COPD: three; stroke: six; Parkinson's disease: two; Alzheimer's disease: two; lumbar spine diseases: three; lower limb fracture: three) and eight were eliminated due to incompleteness of data. After exclusion, a final sample of 292 subjects (139 men; aged 20–89 years) remained. Of them, all but eight were right handed according to the Edinburgh Handedness Inventory [18].

### 2.2. Procedures

Demographic and anthropometric information (age, gender, height and weight) was collected via a health scale (Xiheng®, RGZ-120-RT) prior to participation. Subjects were required to wear comfortable clothes and shoes (not high-heeled shoes or slippers) suitable for the test. Verbal instructions were given: ‘1. Stand still with your feet apart until you hear the long tone; 2. When you hear the tone, start walking at a natural and comfortable pace; 3. When you hear the second tone, stop walking'. After a 30-s practice to be familiar with the test, participants were required to walk back and forth on a 7 m straight walkway (with coloured tapes marking the beginning and the end) at usual comfortable speed (a self-selected natural speed) without walking aids for 2 min. During the test, a number of gait cycles were completed to be used for further analysis. All procedures were conducted by the same experienced investigator, standing nearby to watch the participants for protection.

### 2.3. Apparatus

Data were obtained using a wireless APDM Movement Monitoring inertial sensor system (APDM Inc., Portland, OR, USA). After recalibration, six synchronized Opal inertial sensors were fitted on each participant via elastic straps (sternum, waist (at the level of the fifth lumbar spine, L5), dorsal surface of bilateral wrists and top of each foot). Signals were sampled in accordance with a previous study [19] and streamed to a laptop to be automatically processed and calculated via the corresponding Mobility Lab™ software package.

We chose six gait parameters of lower limbs for further analysis: basic gait parameters (cadence, walking speed, stride length), along with gait cycle duration, ICA and TOA.

### 2.4. Data analysis

Descriptive statistics mean (standard deviation) were applied for all the parameters. All data were analysed with IBM SPSS 22.0 software (IBM Corp, Armonk, New York). We calculated ICC (intraclass correlation coefficient) to evaluate the degree of consistency between right and left gait values. As ICCs were high for all the bilateral values (stride length 0.996, ICA 0.867, TOA 0.883), the variables of the left and right sides were averaged and further used in analysis.

To eliminate the confounding impact of body height, normalization was done to cadence, walking speed and stride length separately for each gender according to Schwesig *et al.* [7].

normalized cadence = cadence × sqrt (body height/mean body height),
normalized speed = speed/sqrt (body height/mean body height),
normalized stride length = stride length/(body height/mean body height).

Normal distribution of the variables was checked with the Kolmogorov–Smirnov test.

Bartlett's test of sphericity and Kaiser–Meyer–Olkin measure of sampling adequacy (KMO) were conducted to assess the suitability for a factor analysis. Principal components factor analysis with varimax rotation was applied to extract factors. The eigenvalues were set to 1.0 as a threshold. Variables with greater absolute values of factor loadings were considered to contribute more to the factor. After the factors were extracted, two-way multivariate analysis of variance (MANOVA) with gender and age group as independent variables was used to analyse data under each factor, and further investigated with *post hoc* Bonferroni corrections for multiple comparisons between age groups. The significance level was set at 0.05.

In addition to study the impact of body mass index (BMI) on each gait parameter, participants were categorized as normal weight (18.5 kg m^−2^ ≤ BMI < 24 kg m^−2^, *n* = 170), overweight (24 kg m^−2^ ≤ BMI < 28 kg m^−2^, *n* = 89) or obese (BMI ≥ 28 kg m^−2^, *n* = 33) according to the Working Group on Obesity in China (WGOC) (2002) [20]. We performed a one-way analysis of covariance (ANCOVA) on each dependent variable (the independent variable: normal weight, overweight and obese) with age and gender as the covariates.

## 3. Results

### 3.1. The demographic and anthropometric information

The study sample was composed of 292 subjects (139 men, 48% and 153 women, 52%) aged between 20 and 89 years (table 1). No differences were found between the ages of men and women overall and in each age group. Height ((M--W)/W = 8.07%, *p* < 0.001) and weight ((M--W)/W = 19.10%, *p* < 0.001) were significantly higher in men than women. BMI values were comparable for men and women, except for the subjects in the 20–29 ((M--W)/W = 9.69%, *p* < 0.01) and 30–39 ((M--W)/W = 8.60%, *p* < 0.05) age groups, where men's values were greater than women's.

**Table 1.** Descriptive characteristics of participants (*n* = 292) grouped by age and gender. All variables are presented as mean (standard deviation); BMI, body mass index; 95% CI (M--W), 95% confidence Interval of mean difference between genders. *p < 0.05, **p < 0.01, ***p < 0.001, statistical significance between genders.

age groups (years)	men/women	age (years)	height (cm)	weight (kg)	BMI (kg m^−2^)
all participants					
20–89	M (*n* = 139)	52.76 (19.37)	171.63 (5.87)***	70.54 (10.26)***	23.92 (3.14)
	W (*n* = 153)	55.93 (19.54)	158.81 (5.18)	59.23 (9.17)	23.48 (3.49)
	95% CI (M--W)	−7.65 to 1.32	11.55–14.10	9.07–13.55	−0.33 to 1.21
participants by age intervals					
20–29	M (*n* = 21)	23.86 (2.52)	175.76 (5.22)***	68.33 (9.60)***	22.08 (2.64)**
	W (*n* = 20)	24.60 (2.33)	164.10 (4.22)	54.20 (4.41)	20.13 (1.49)
	95% CI (M--W)	−2.28 to 0.79	8.65–14.67	9.39–18.87	0.58–3.31
30–39	M (*n* = 21)	35.29 (2.72)	173.05 (6.64)***	72.26 (10.11)***	24.11 (3.03)*
	W (*n* = 20)	35.05 (3.12)	160.25 (3.01)	57.08 (6.83)	22.20 (2.36)
	95% CI (M--W)	−1.61 to 2.08	9.53–16.07	9.74–20.63	0.20–3.64
40–49	M (*n* = 21)	44.76 (2.93)	170.57 (4.01)***	73.02 (11.23)***	25.09 (3.68)
	W (*n* = 21)	46.33 (2.63)	157.24 (3.62)	58.52 (6.69)	23.66 (2.48)
	95% CI (M--W)	−3.31 to 0.17	10.95–15.71	8.70–20.30	−0.54 to 3.39
50–59	M (*n* = 21)	54.86 (2.92)	169.67 (5.10)***	70.86 (11.01)**	24.52 (3.00)
	W (*n* = 24)	56.42 (2.80)	159.04 (4.89)	61.67 (10.55)	24.36 (3.99)
	95% CI (M--W)	−3.28 to 0.16	7.62–13.63	2.70–15.68	−1.99 to 2.30
60–69	M (*n* = 23)	64.13 (3.39)	171.04 (4.69)***	72.98 (9.27)**	24.90 (2.55)
	W (*n* = 28)	65.75 (2.46)	158.50 (5.12)	64.13 (11.09)	25.47 (3.95)
	95% CI (M--W)	−3.33 to 0.09	9.76–15.33	3.02–14.68	−2.49 to 1.35
70–89	M (*n* = 32)	78.91 (4.55)	170.41 (6.83)***	67.25 (9.88)***	23.15 (3.08)
	W (*n* = 40)	79.90 (5.75)	156.35 (5.44)	58.29 (9.18)	23.80 (3.22)
	95% CI (M--W)	−3.42 to 1.43	11.17–16.94	4.47–13.45	−2.14 to 0.84

### 3.2. Factor analysis

Table 2 displays the correlation coefficient matrix of the gait variables (calculated as Pearson's correlation coefficient, R). It can be clearly observed that correlation coefficients of many variables were considerably large and the corresponding *p*-values were statistically significant, implying that there were significant correlations between these variables necessary for a factor analysis. Note that both ICA and TOA were highly correlated with normalized speed (0.674 and 0.772, respectively) and normalized stride length (0.815 and 0.764, respectively).

**Table 2.** Pearson's correlation coefficient matrix of gait variables. Values in bold indicate highly positive correlations of ICA and TOA with normalized speed and normalized stride length.

gait	normalized	normalized	initial contact	toe-off	normalized	gait cycle
variable	speed	stride length	angle	angle	cadence	duration
normalized speed	1.000	0.910	0.674	0.772	0.650	−0.648
normalized stride length	0.910	1.000	0.815	0.764	0.287	−0.303
initial contact angle	**0**.**674**	**0**.**815**	1.000	0.511	0.085	−0.101
toe-off angle	**0**.**772**	**0**.**764**	0.511	1.000	0.412	−0.418
normalized cadence	0.650	0.287	0.085	0.412	1.000	−0.975
gait cycle duration	−0.648	−0.303	−0.101	−0.418	−0.975	1.000
*p*-value						
normalized speed		<0.001	<0.001	<0.001	<0.001	<0.001
normalized stride length	<0.001		<0.001	<0.001	<0.001	<0.001
initial contact angle	<0.001	<0.001		<0.001	0.08	<0.05
toe-off angle	<0.001	<0.001	<0.001		<0.001	<0.001
normalized cadence	<0.001	<0.001	0.08	<0.001		<0.001
gait cycle duration	<0.001	<0.001	<0.05	<0.001	<0.001	

In this research, the Bartlett's and KMO test values were 0.000 and 0.648, respectively, implying factor analysis was suitable for six gait variables. Two factors extracted by factor analysis could explain 90.2% of the variance. The first one labelled as a ‘Progress’ factor accounted for 50.8% of the total variance and loaded highly on normalized speed, normalized stride length, ICA and TOA. The second one labelled as a ‘Rhythm’ factor accounted for 39.4% of the total variance and had the high loadings of normalized cadence and gait cycle duration. Factor loadings of gait parameters on two factors were shown in table 3.

**Table 3.** Varimax rotated factor loadings of gait parameters by factor analysis (two factors extracted).

gait parameters	Progress	Rhythm
normalized speed	0.819	0.546
normalized stride length	0.964	0.166
initial contact angle	0.903	−0.085
toe-off angle	0.766	0.354
normalized cadence	0.140	0.980
gait cycle duration	−0.152	−0.975

### 3.3. Age, gender and interaction effects on gait parameters and *post hoc* comparisons of age groups by MANOVA

For the first factor of ‘Progress’, the gender (*F*_4,277_ = 56.073, *p* < 0.001) and age group (*F*_20,1120_ = 9.014, *p* < 0.001) main effects were significant, but the gender × age interaction was not (*F*_20,1120_ = 1.213, *p* = 0.23). With respect to the second factor of ‘Rhythm', the gender main effect (*F*_2,279_ = 17.431, *p* < 0.001), age group main effect (*F*_10,560_ = 8.388, *p* < 0.001) and the gender × age interaction (*F*_10,560_ = 1.999, *p* = 0.03) were all statistically significant.

Concerning each gait parameter, MANOVA showed significant main effects of both gender and age (gender effect on normalized speed at *p* < 0.01 and the others *p* < 0.001) except for the gender effect on TOA (*p* = 0.09). However, gender × age interaction effect failed to achieve statistical significance with the exception of ICA (*p* = 0.04).

Reference data of gait parameters comprising the two factors by age and gender are shown in table 4.

**Table 4.** Normative reference data of gait parameters composing the two factors ‘Progress’ and ‘Rhythm’ by age and gender. Data are presented as mean (standard deviation).

		age groups by years					
gait parameters	men/women	20–29	30–39	40–49	50–59	60–69	70–89
normalized speed (m s^−1^)	M	1.17 (0.10)	1.19 (0.16)	1.23 (0.16)	1.25 (0.18)	1.12 (0.13)	0.87 (0.25)
	W	1.11 (0.14)	1.13 (0.12)	1.06 (0.11)	1.19 (0.09)	1.14 (0.12)	0.85 (0.22)
	total	1.14 (0.12)	1.16 (0.14)	1.14 (0.16)	1.21 (0.14)	1.13 (0.12)	0.86 (0.23)
normalized stride length (m)	M	1.28 (0.12)	1.29 (0.12)	1.32 (0.10)	1.32 (0.15)	1.23 (0.12)	1.01 (0.24)
	W	1.15 (0.09)	1.16 (0.07)	1.12 (0.09)	1.18 (0.08)	1.15 (0.10)	0.92 (0.19)
	total	1.22 (0.12)	1.23 (0.12)	1.22 (0.14)	1.25 (0.13)	1.19 (0.11)	0.96 (0.22)
initial contact angle (°)	M	27.57 (4.98)	26.44 (5.77)	26.80 (4.07)	24.72 (6.52)	22.67 (4.61)	16.80 (6.76)
	W	20.18 (4.09)	20.06 (3.23)	18.77 (4.06)	18.62 (3.85)	19.43 (4.38)	13.73 (4.87)
	total	23.97 (5.86)	23.33 (5.66)	22.79 (5.71)	21.47 (6.05)	20.89 (4.73)	15.10 (5.94)
toe-off angle (°)	M	69.97 (4.28)	67.23 (6.71)	69.69 (5.16)	69.94 (9.02)	64.83 (7.56)	53.56 (11.65)
	W	70.41 (6.13)	70.07 (6.14)	69.15 (7.72)	71.76 (5.37)	67.64 (7.27)	56.43 (11.84)
	total	70.18 (5.20)	68.61 (6.52)	69.42 (6.49)	70.91 (7.27)	66.37 (7.46)	55.16 (11.76)
normalized cadence (steps min^−1^)	M	109.88 (6.46)	110.07 (8.93)	111.32 (9.14)	113.09 (8.82)	108.55 (6.59)	101.88 (10.38)
	W	114.91 (9.21)	116.40 (8.18)	112.44 (6.04)	119.48 (5.20)	118.80 (7.57)	107.82 (10.60)
	total	112.33 (8.22)	113.16 (9.05)	111.88 (7.67)	116.50 (7.74)	114.18 (8.76)	105.18 (10.85)
gait cycle duration (s)	M	1.11 (0.06)	1.10 (0.10)	1.08 (0.09)	1.06 (0.08)	1.11 (0.07)	1.19 (0.12)
	W	1.07 (0.09)	1.04 (0.08)	1.07 (0.06)	1.01 (0.05)	1.01 (0.06)	1.12 (0.12)
	total	1.09 (0.08)	1.07 (0.09)	1.07 (0.07)	1.03 (0.07)	1.06 (0.08)	1.15 (0.13)

After normalization, men walked with larger stride length (mean difference(M--W) = 0.13 m, *p* < 0.001, 95% CI = 0.09–0.16 m), faster speed (mean difference(M--W) = 0.06 m s^−1^, *p* < 0.01, 95% CI = 0.02–0.10 m s^−1^), longer gait cycle (mean difference(M--W) = 0.06 s, *p* < 0.001, 95% CI = 0.04–0.08 s), but decreased cadence (mean difference(M--W) = −5.84 steps min^−1^, *p* < 0.001, 95% CI = −7.84 to −3.85 steps min^−1^) than did women. Larger ICAs were found in men (mean difference(M--W) = 5.70°, *p* < 0.001, 95% CI = 4.54–6.87°), whereas larger TOAs were observed in women (mean difference(M--W) = −1.71°, *p* = 0.09, 95%CI = −3.65–0.24°) but did not reach statistical significance.

Increasing age was associated with progressively smaller ICAs. Gait cycle duration prolonged, and normalized cadence and normalized speed reduced evidently in the oldest age group (70--89 years). Normalized stride length and TOA declined after 60 years old. Owing to *post hoc* Bonferroni's corrections, only the oldest age group (70–89 years) showed significantly lower values of all gait variables compared with the other groups.

### 3.4. The influence of body mass index on the gait parameters by ANCOVA

ANCOVA conducted with age and gender as covariates showed the impact of BMI on gait performance. Overweight and obese adults walked at significantly lower speed (mean difference(normal–overweight) = 0.08 m s^−1^, *p* < 0.01, 95% CI = 0.02–0.14 m s^−1^ and mean difference(normal–obese) =0.15 m s^−1^, *p* < 0.001, 95% CI = 0.07–0.24 m s^−1^), shorter stride length (mean difference(normal–overweight) = 0.06 m, *p* = 0.01, 95% CI = 0.01–0.11 m and mean difference(normal–obese) = 0.11 m, *p* < 0.01, 95% CI = 0.03–0.18 m), reduced cadence (mean difference(normal–overweight) = 3.15 steps min^−1^, *p* = 0.03, 95% CI = 0.23–6.07 steps min^−1^ and mean difference(normal–obese) = 6.56 steps min^−1^, *p* = 0.001, 95% CI = 2.30–10.83 steps min^−1^) and longer gait cycle duration (mean difference(normal–overweight) = −0.04 s, *p* = 0.02, 95% CI = −0.07 to −0.004 s and mean difference(normal–obese) = −0.07 s, *p* = 0.001, 95% CI = −0.12 to −0.03 s) compared with the normal weight group. TOA was smaller in the obese group than in normal weight adults (mean difference(normal–obese) = 6.23°, *p* = 0.001, 95% CI = 1.98–10.49°). However, the differences in ICAs did not reach statistical significance. No significant differences were found in all variables between the overweight and the obese group (table 5).

**Table 5.** The influence of BMI on each gait parameter, while controlling for age and gender by ANCOVA. *p < 0.05, **p < 0.01, ***p < 0.001, statistical significance compared to normal weight group. BMI, body mass index; 95% CI, 95% confidence interval.

	values adjusted by age and gender								
	normal weight (*n* = 170)		overweight (*n* = 89)		obese (*n* = 33)		95% CI of mean difference		
gait parameters	mean	95% CI	mean	95% CI	mean	95% CI	normal–overweight	normal–obese	overweight–obese
normalized speed (m s^−1^)	1.13	1.10– 1.16	1.05**	1.01–1.08	0.97***	0.91–1.04	0.02–0.14	0.07–0.24	−0.02 to 0.16
normalized stride length (m)	1.19	1.16–1.21	1.12*	1.09–1.16	1.08**	1.02–1.13	0.01–0.11	0.03– 0.18	−0.03 to 0.12
initial contact angle (°)	21.14	20.34–21.95	19.82	18.72–20.92	19.90	18.07–21.73	−0.38 to 3.03	−1.25 to 3.73	−2.64 to 2.48
toe-off angle (°)	67.16	65.79–68.54	64.33	62.45–66.21	60.93**	57.81–64.05	−0.08 to 5.73	1.98–10.49	−0.97 to 7.78
normalized cadence (steps min^−1^)	113.29	111.91–114.67	110.14*	108.25–112.02	106.72**	103.60–109.85	0.23–6.07	2.30–10.83	−0.97 to 7.80
gait cycle duration (s)	1.07	1.05–1.08	1.10*	1.08–1.12	1.14**	1.10–1.17	−0.07 to −0.004	−0.12 to −0.03	−0.08 to 0.01

## 4. Discussion

Normative group data provided in this study are of great importance to interpret and evaluate normal gait performance and disorders. Different from the conventional 2MWT [21], which measures the farthest possible distance covered in 2 min, the walk test in this study did not measure distance, but did provide many useful gait metrics. Given that there would be some variability in distance as the software recognizes and processes information from turns, the algorithms in Mobility Lab do not measure distance. We used a natural and comfortable speed that could best reflect the natural walking performance of daily life and was suitable for establishing a reference database. Technically, Mobility Lab requires a minimum of three gait cycles to determine the normal gait rate of the subject. We set the test condition of 2 min for the reason that it included adequate gait cycles to produce stable and reliable results. Moreover, a walk of 2 min was a sustainable energy consumption for most clinical patients [22–24] for further comparisons.

Similar to Hollman *et al*. [6], we identified a ‘Rhythm' factor that included normalized cadence and gait cycle duration. While Hollman *et al*. [6] included gait speed and stride length in a ‘Pace' factor, our study suggested that two additional parameters of ICA and TOA also loaded highly on the factor and named it ‘Progress’. The correlation coefficient matrix demonstrated highly positive correlations of ICA and TOA with normalized speed and normalized stride length. The underlying mechanism may be partly explained by a unified prospective on ankle push-off proposed by Zelik *et al*. [25], that ankle push-off contributed to both leg swing and to center of mass acceleration during walking, manifesting principally as increased speed and kinetic energy of the push-off limb. Fong *et al*. [26] found that humans use greater toe grip and gentler heel strike to prevent slip. By adopting these two strategies, the ground reaction force shifted towards a more vertical direction, flat foot landing was attained and ground friction force increased. Cham *et al*. [27] observed smaller heel contact foot--floor angles in subjects who recovered from slip events on oily surfaces compared with on dry conditions. Similar results were observed during falling trials. These were in accordance with our study that ICA gradually decreased with increasing age, especially in the elderly, to keep balance and achieve safe walking. Ankle dorsiflexion restriction derived from soft tissue or bony tissue factors at initial contact was shown to be related to greater vertical ground reaction forces (vGRF) which increased knee joint loading [28,29]. Subjects with early signs of knee osteoarthrosis had a higher loading rate of the vGRF associated with heel strike than age-matched controls [30]. Measures modifying restricted ankle dorsiflexion during walking may serve as a means to prevent early knee osteoarthrosis. Older adults showed reduced push-off reactions of the supporting limb and less proper placement of the recovery limb in recovery after tripping, particularly in older fallers. This was ascribed to a lower peak ankle moment and a lower rate of change of moment generation in all supporting limb joints. Lower limb strength could be an underlying factor [31]. In the elderly, ankle strength training to increase ICA and TOA during walking might help to prevent falls after tripping and improve gait ability. This needs investigation in future work.

Several factors could account for different gait values from different studies, including specific ethnic groups studied, test items and environments, and technical equipment. In our study, gait values were obtained from a walk test for 2 min by the APDM Movement Monitoring inertial sensor system, the reliability and validity of which have already been verified [19,32]. Hollman *et al*. [33], also using APDM to collect data, reported a mean stride length of 1.57 ± 0.09 m, a mean stride time of 1.03 ± 0.05 s, a mean stride velocity of 1.53 ± 0.10 m s^−1^ and a mean cadence of 116.4 ± 5.6 steps min^−1^ in young adults (nine men, 11 women, 23.8 ± 1.2 years old) along a 42 m path for 6 min. The same age group in our study, however, showed a shorter mean stride length(1.22 ± 0.12 m), lower gait speed (1.14 ± 0.12 m s^−1^), slightly longer gait cycle duration (1.09 ± 0.08 s) and slightly lower cadence (112.33 ± 8.22 steps min^−1^). These differences may be attributed partly to the length of the walkway, the test time and the greater height and weight of the subjects in the Hollman study compared with our study. Moreover, normalization of gait parameters was not conducted in the Hollman study. In an acceleration-based gait test using similarly aged subjects (20–86 years old), Senden *et al*. [8] reported significantly larger steps (0.80 ± 0.08 m versus 0.71 ± 0.07 m), longer step time (0.55 ± 0.03 s versus 0.51 ± 0.03 s), greater speed (1.49 ± 0.20 m s^−1^ versus 1.40 ± 0.17 m s^−1^) and lower cadence (110.51 ± 6.30 steps min^−1^ versus 118.43 ± 6.94 steps min^−1^) in men than women. When scaled for leg length, the speed showed similar values between genders. Our findings were similar. Stride length in men exceeded that in women by 0.13 m (95%CI = 0.09–0.16 m) and gait speed in men exceeded that in women by 0.06 m s^−1^ (95%CI = 0.02–0.10 m s^−1^). Gait cycle duration in men exceeded that in women by 0.06 s (95%CI = 0.04–0.08 s), but cadence was lower in men than in women by −5.84 steps min^−1^ (95%CI = −7.84 to −3.85 steps min^−1^). After normalization, walking speeds in our study were comparable in men and women in most age groups. However, gender effect was revealed in normalized speed. This may result from the large difference between men and women (1.23 ± 0.16 m s^−1^ versus 1.06 ± 0.11 m s^−1^) in the 40–49 years age group. Our findings were in general agreement with Frimenko *et al*. [34], who indicated men and women applied different strategies to achieve similar speed: larger step length in men and higher cadence in women. Old age is an important transition period in mobility function. As illustrated in this study, gait performance decreased obviously after the age of 70. This was consistent with a previous study [35] that found walking speed reduced significantly in the elderly. Gait dysfunction in older adults was found to be associated with several health problems, for example, increased risk of fall, weakness, injury, cognitive impairment and higher incidence of dementia [36,37]. The selected gait variables in our study may reflect gait impairments in old adults to some extent; however, their relationships with other health problems need further investigation.

BMI had a significant impact on gait variables in the ‘Progress’ factor, with decreased speed and stride length in overweight and obese adults compare with the normal weight group, as well as less TOA of the obese group compared to the normal weight group. ICAs were not significantly different between all three groups. With regard to the ‘Rhythm' factor, less cadence and more gait cycle duration were found in overweight and obese groups. These results suggested that age, gender and BMI factors should be considered in the reference gait metrics to help identify gait abnormalities and pathologies more accurately. Overweight and obesity are known to exert important influence on the locomotor system. Obesity is relevant to increased loading of lower limbs, gait alteration and decreased motor ability [38,39]. For example, obese adults were more likely to have a heel strike transient than normal weight persons, which indicated a rapid rise in vGRF ensued after ground contact to produce high vGRF loading rate that increased the risk of knee osteoarthritis [40]. As shown in the current study, gait altered in overweight and obese adults with less TOA, speed, stride length and cadence, and increased gait cycle duration. Especially for old adults, weight loss by having a healthy diet and regular exercise will favourably modify gait and balance, prevent knee injury and improve quality of life.

Several limitations should be mentioned. First, using the subject's leg length instead of body height might be more appropriate for normalization of gait parameters. For instance, body height reduced from kyphosis would not affect one's gait performance as leg length does. Second, some interpretations of kinematic outcome measures relied on evidence from studies of GRF, joint moments and power estimates. These might not be fair comparisons. Third, only gait parameters of lower limbs were investigated in this study. Parameters of trunk and upper limbs should be examined in future work to improve our knowledge of gait function. Also, future studies need to include more subjects to yield more stable results.

## 5. Conclusion

The normative reference database established can be applied for future comparisons in a wide range of clinical patients with gait pathologies and abnormalities using the APDM Movement Monitoring inertial sensor system, thus contributing to the assessment and identification of mobility dysfunction as well as the efficacy of rehabilitation interventions.

## Supplementary Material

ICC for all the bilateral values.; The effects of gender, age, and gender×age interaction on each variable.; The reference data of this manuscript.

## Data accessibility

Gait data supporting this article have been uploaded as the electronic supplementary material.
