# Supplementary material for: Reference values of gait using APDM movement monitoring inertial sensor system
Source: R Soc Open Sci. 2018 Jan 10;5(1):170818. doi: 10.1098/rsos.170818 (PMC5792878; doi:10.1098/rsos.170818)
Supplement: ICC for all the bilateral values.; The effects of gender, age, and gender×age interaction on each variable.; The reference data of this manuscript. [file rsos170818supp1.doc]

**Royal Society Open Science**

**Reference Values of Gait Using APDM Movement Monitoring Inertial Sensor System**

Xin Fang^a,1^, Chuandao Liu^b,1^, Zhongli Jiang^a,*^

^a^ Department of Rehabilitation Medicine, The First Affiliated Hospital of Nanjing Medical University, Nanjing, 210029, China

^b^ Department of Rehabilitation, the First Affiliated Hospital of Soochow University, Suzhou, 215006, China

* Author for correspondence: E-mail address: jiangzh3721@163.com

^1^ These authors contributed equally to this work.

Table S1. ICC for all the bilateral values. ICC=Intraclass Correlation Coefficient, 95% CI=95% Confidence Interval.

| Gait Parameters | ICC | 95% CI | F-value | P-value |
| --- | --- | --- | --- | --- |
| Stride Length (m) | 0.996 | 0.995 to 0.997 | 506.146 | <.001 |
| Initial Contact Angle (°) | 0.867 | 0.836 to 0.893 | 14.070 | <.001 |
| Toe-Off Angle (°) | 0.883 | 0.855 to 0.906 | 16.106 | <.001 |

Table S2. The effects of gender, age and gender×age interaction on each variable.

|  |  | F-value | P-value |
| --- | --- | --- | --- |
| Gender | Normalized Speed | 9.328 | .002 |
|  | Normalized Stride Length | 55.899 | .000 |
|  | Initial Contact Angle | 92.780 | .000 |
|  | Toe-Off Angle | 2.981 | **.085** |
|  | Normalized Cadence | 33.200 | .000 |
|  | Gait Cycle Duration | 27.749 | .000 |
| Age | Normalized Speed | 38.475 | .000 |
|  | Normalized Stride Length | 36.248 | .000 |
|  | Initial Contact Angle | 24.587 | .000 |
|  | Toe-Off Angle | 32.603 | .000 |
|  | Normalized Cadence | 12.717 | .000 |
|  | Gait Cycle Duration | 12.170 | .000 |
| Gender × Age | Normalized Speed | 1.908 | .093 |
|  | Normalized Stride Length | 1.095 | .363 |
|  | Initial Contact Angle | 2.315 | **.044** |
|  | Toe-Off Angle | .354 | .879 |
|  | Normalized Cadence | 1.369 | .236 |
|  | Gait Cycle Duration | 1.080 | .371 |

Table S3. The reference data of this manuscript.

| Subject | Gender | Age group  (years) | Normalized  Speed (m/s) | Normalized  Stride Length (m) | Initial Contact  Angle (°) | Toe-Off Angle (°) | Normalized  Cadence  (steps/min) | Gait Cycle  Duration (s) |
| --- | --- | --- | --- | --- | --- | --- | --- | --- |
| 1 | male | 20~29 | 1.117336 | 1.215152 | 23.59585 | 76.5404 | 108.8361 | 1.10421 |
| 2 | male |  | 1.142362 | 1.19971 | 29.0162 | 65.21355 | 113.3324 | 1.03936 |
| 3 | male |  | 1.242641 | 1.410525 | 28.6665 | 69.75475 | 104.2892 | 1.17218 |
| 4 | male |  | 1.196873 | 1.208457 | 22.7933 | 66.65885 | 117.932 | 1.04864 |
| 5 | male |  | 0.962299 | 1.093535 | 21.0447 | 64.02505 | 104.9292 | 1.13915 |
| 6 | male |  | 1.21712 | 1.401502 | 35.27095 | 71.26915 | 104.1306 | 1.16408 |
| 7 | male |  | 1.100251 | 1.113789 | 21.6676 | 64.141 | 117.6448 | 1.04526 |
| 8 | male |  | 1.174995 | 1.303803 | 27.957 | 72.68705 | 107.6344 | 1.14836 |
| 9 | male |  | 1.097517 | 1.213186 | 36.46075 | 67.0967 | 108.1349 | 1.08857 |
| 10 | male |  | 1.386877 | 1.339777 | 30.11655 | 71.8328 | 122.9408 | 0.988867 |
| 11 | male |  | 1.224191 | 1.229261 | 29.42565 | 63.7823 | 119.276 | 1.01624 |
| 12 | male |  | 1.24903 | 1.42436 | 32.01955 | 75.5829 | 104.2778 | 1.16588 |
| 13 | male |  | 1.26015 | 1.458719 | 33.4042 | 67.67725 | 103.0905 | 1.1759 |
| 14 | male |  | 1.177956 | 1.355443 | 31.0332 | 73.71725 | 102.9365 | 1.17477 |
| 15 | male |  | 1.197511 | 1.395009 | 32.72855 | 73.7698 | 103.4159 | 1.1884 |
| 16 | male |  | 1.13452 | 1.202421 | 23.04255 | 72.18335 | 112.5547 | 1.06183 |
| 17 | male |  | 1.31377 | 1.348377 | 22.5357 | 73.40045 | 115.0078 | 1.08443 |
| 18 | male |  | 1.246089 | 1.359534 | 27.16665 | 76.40365 | 109.279 | 1.12479 |
| 19 | male |  | 1.037239 | 1.189618 | 25.87015 | 66.77835 | 103.9282 | 1.18461 |
| 20 | male |  | 1.038651 | 1.042946 | 18.32485 | 65.0298 | 118.8306 | 1.0291 |
| 21 | male |  | 1.144107 | 1.300745 | 26.7813 | 71.8688 | 105.0535 | 1.15825 |
| 22 | male | 30~39 | 1.133774 | 1.209676 | 28.34345 | 61.2726 | 112.0425 | 1.0757 |
| 23 | male |  | 0.857898 | 1.124116 | 18.24895 | 53.47915 | 91.65103 | 1.30976 |
| 24 | male |  | 1.42166 | 1.428437 | 23.8651 | 73.42035 | 119.4745 | 1.00071 |
| 25 | male |  | 1.260743 | 1.224187 | 17.90185 | 70.75075 | 123.0818 | 1.00456 |
| 26 | male |  | 1.090827 | 1.133941 | 22.05285 | 64.35475 | 115.0979 | 1.0774 |
| 27 | male |  | 1.200668 | 1.17001 | 26.84055 | 60.2505 | 122.4595 | 0.963965 |
| 28 | male |  | 1.286133 | 1.316547 | 23.61215 | 69.8277 | 115.7991 | 1.03187 |
| 29 | male |  | 1.227786 | 1.256889 | 21.21085 | 62.0161 | 116.6233 | 1.03947 |
| 30 | male |  | 0.931636 | 1.143014 | 20.90895 | 75.8637 | 97.0876 | 1.27113 |
| 31 | male |  | 1.201087 | 1.361041 | 29.507 | 67.2123 | 104.9526 | 1.20415 |
| 32 | male |  | 1.341116 | 1.357229 | 29.1354 | 70.11425 | 117.1536 | 1.02008 |
| 33 | male |  | 1.366004 | 1.359667 | 22.21595 | 79.7271 | 120.8521 | 1.01172 |
| 34 | male |  | 1.139343 | 1.354079 | 31.08365 | 68.6749 | 100.0655 | 1.19451 |
| 35 | male |  | 1.118977 | 1.285963 | 27.7093 | 68.8024 | 104.0029 | 1.14625 |
| 36 | male |  | 1.346501 | 1.416589 | 26.65685 | 70.7608 | 113.2682 | 1.05578 |
| 37 | male |  | 1.363392 | 1.466272 | 33.43925 | 66.99185 | 109.7374 | 1.09884 |
| 38 | male |  | 1.314054 | 1.401829 | 33.77725 | 78.1482 | 111.8994 | 1.03682 |
| 39 | male |  | 1.18229 | 1.426933 | 32.8562 | 67.5312 | 99.74195 | 1.21709 |
| 40 | male |  | 1.287817 | 1.377163 | 40.53395 | 59.03095 | 110.8835 | 1.07506 |
| 41 | male |  | 0.913319 | 1.074825 | 23.70815 | 57.73215 | 100.3107 | 1.20404 |
| 42 | male |  | 1.001777 | 1.13333 | 21.72445 | 65.93215 | 105.2174 | 1.13075 |
| 43 | male | 40~49 | 1.211015 | 1.36047 | 29.77715 | 79.23725 | 106.3972 | 1.12315 |
| 44 | male |  | 1.237715 | 1.329986 | 25.1218 | 65.44885 | 111.0826 | 1.07966 |
| 45 | male |  | 1.348732 | 1.307188 | 24.7541 | 70.93435 | 122.9797 | 0.971512 |
| 46 | male |  | 1.378442 | 1.381334 | 32.9236 | 67.1549 | 118.8926 | 1.01406 |
| 47 | male |  | 1.422654 | 1.461811 | 30.2809 | 73.44565 | 117.1384 | 1.03203 |
| 48 | male |  | 1.203941 | 1.307415 | 27.2571 | 70.70865 | 109.7431 | 1.08874 |
| 49 | male |  | 1.262702 | 1.457342 | 27.7562 | 71.27815 | 102.2381 | 1.16905 |
| 50 | male |  | 1.027968 | 1.265145 | 29.43055 | 72.0774 | 97.20952 | 1.22239 |
| 51 | male |  | 1.332367 | 1.381657 | 24.26095 | 70.08845 | 113.9099 | 1.04309 |
| 52 | male |  | 1.219638 | 1.280322 | 25.0435 | 69.2645 | 113.344 | 1.04776 |
| 53 | male |  | 1.129409 | 1.274043 | 25.80875 | 69.1489 | 106.3739 | 1.10818 |
| 54 | male |  | 1.266882 | 1.253598 | 34.21935 | 67.0233 | 120.3433 | 0.993011 |
| 55 | male |  | 0.869605 | 1.159912 | 29.39035 | 59.94815 | 89.26581 | 1.32133 |
| 56 | male |  | 1.117531 | 1.251747 | 26.5654 | 69.62015 | 105.9604 | 1.12151 |
| 57 | male |  | 1.314136 | 1.426918 | 29.73195 | 70.2977 | 110.0994 | 1.08882 |
| 58 | male |  | 1.536397 | 1.511285 | 17.11225 | 82.3864 | 121.1097 | 0.986503 |
| 59 | male |  | 1.355755 | 1.277616 | 22.4436 | 73.01065 | 126.3458 | 0.981975 |
| 60 | male |  | 0.936011 | 1.104662 | 21.84755 | 60.9685 | 101.0915 | 1.18111 |
| 61 | male |  | 1.215885 | 1.29723 | 25.3682 | 70.4389 | 111.1125 | 1.08141 |
| 62 | male |  | 1.194587 | 1.26435 | 31.19795 | 63.75795 | 113.2454 | 1.05529 |
| 63 | male |  | 1.324173 | 1.32547 | 22.54435 | 67.3184 | 119.905 | 1.01997 |
| 64 | male | 50~59 | 1.285989 | 1.346903 | 25.8446 | 75.9497 | 114.5916 | 1.05212 |
| 65 | male |  | 1.567035 | 1.450043 | 26.57865 | 76.90305 | 126.9037 | 0.933325 |
| 66 | male |  | 1.205012 | 1.317754 | 22.52055 | 77.2983 | 108.6406 | 1.09334 |
| 67 | male |  | 1.130919 | 1.22674 | 24.6552 | 81.5916 | 110.7139 | 1.05683 |
| 68 | male |  | 1.167283 | 1.276995 | 14.63875 | 67.63015 | 107.809 | 1.11574 |
| 69 | male |  | 1.234101 | 1.19639 | 16.6644 | 72.4902 | 122.8606 | 0.967119 |
| 70 | male |  | 0.913459 | 1.113882 | 21.59205 | 53.9985 | 96.38473 | 1.19677 |
| 71 | male |  | 1.24772 | 1.420592 | 23.24955 | 71.33645 | 105.2537 | 1.13862 |
| 72 | male |  | 1.229422 | 1.209237 | 17.03215 | 66.11035 | 121.5626 | 0.977019 |
| 73 | male |  | 1.355019 | 1.268334 | 23.4399 | 69.2974 | 127.7932 | 0.94581 |
| 74 | male |  | 1.346611 | 1.369996 | 30.1405 | 70.12145 | 117.6736 | 1.02161 |
| 75 | male |  | 1.299675 | 1.422079 | 25.0535 | 82.6449 | 110.6681 | 1.08916 |
| 76 | male |  | 1.099935 | 1.234968 | 20.8808 | 56.07705 | 107.1175 | 1.1391 |
| 77 | male |  | 1.157181 | 1.374734 | 32.5362 | 69.61755 | 100.0527 | 1.17664 |
| 78 | male |  | 1.544032 | 1.591868 | 40.06705 | 78.52195 | 116.9252 | 1.04587 |
| 79 | male |  | 1.416926 | 1.369542 | 27.28455 | 77.8358 | 123.3797 | 0.974837 |
| 80 | male |  | 1.064163 | 1.121985 | 19.56145 | 57.31335 | 113.9643 | 1.04233 |
| 81 | male |  | 1.3433 | 1.278682 | 21.02265 | 69.48605 | 123.0173 | 0.951341 |
| 82 | male |  | 1.518235 | 1.711305 | 37.5163 | 79.1474 | 107.2664 | 1.10765 |
| 83 | male |  | 1.078302 | 1.235826 | 29.50165 | 61.8199 | 105.0163 | 1.16454 |
| 84 | male |  | 0.992386 | 1.106298 | 19.4144 | 53.4907 | 107.3564 | 1.10736 |
| 85 | male | 60~69 | 1.028445 | 1.18549 | 22.15325 | 63.68005 | 103.4184 | 1.15571 |
| 86 | male |  | 0.799763 | 0.865015 | 14.01375 | 40.03335 | 108.3201 | 1.12166 |
| 87 | male |  | 1.24338 | 1.248125 | 21.26495 | 59.20545 | 117.4192 | 1.0115 |
| 88 | male |  | 1.067258 | 1.122084 | 19.66105 | 61.76415 | 113.5002 | 1.05006 |
| 89 | male |  | 1.238713 | 1.226688 | 23.99855 | 64.30695 | 121.3137 | 0.97642 |
| 90 | male |  | 1.309826 | 1.349728 | 28.87675 | 68.552 | 114.9392 | 1.03949 |
| 91 | male |  | 1.100577 | 1.190638 | 20.59535 | 60.0828 | 109.8115 | 1.10401 |
| 92 | male |  | 0.953152 | 1.067235 | 12.54035 | 59.2559 | 105.5741 | 1.13165 |
| 93 | male |  | 1.09965 | 1.176665 | 19.5192 | 60.8058 | 112.1945 | 1.06804 |
| 94 | male |  | 1.198978 | 1.245223 | 22.97755 | 78.922 | 114.5461 | 1.04346 |
| 95 | male |  | 0.99884 | 1.226854 | 18.20325 | 61.3188 | 97.02655 | 1.21689 |
| 96 | male |  | 1.170406 | 1.338585 | 24.13525 | 74.50485 | 105.0596 | 1.16719 |
| 97 | male |  | 1.213941 | 1.364453 | 27.52795 | 69.66595 | 106.6627 | 1.12371 |
| 98 | male |  | 1.218308 | 1.304431 | 27.3416 | 64.2379 | 110.9013 | 1.08423 |
| 99 | male |  | 1.182549 | 1.308253 | 22.3649 | 66.48825 | 108.7977 | 1.09974 |
| 100 | male |  | 1.101668 | 1.29028 | 25.8658 | 68.12495 | 102.9001 | 1.14503 |
| 101 | male |  | 1.233508 | 1.330404 | 28.89645 | 64.8296 | 110.7454 | 1.08867 |
| 102 | male |  | 1.027852 | 1.251899 | 25.8892 | 67.35845 | 98.96181 | 1.21593 |
| 103 | male |  | 1.13809 | 1.177168 | 18.09725 | 77.2869 | 115.4068 | 1.02906 |
| 104 | male |  | 1.229694 | 1.318676 | 22.94535 | 67.0026 | 111.8024 | 1.09962 |
| 105 | male |  | 0.905851 | 1.152016 | 18.87455 | 63.51085 | 93.95513 | 1.30891 |
| 106 | male |  | 1.09457 | 1.248596 | 29.9056 | 63.3812 | 105.4273 | 1.14443 |
| 107 | male |  | 1.269276 | 1.39107 | 25.7137 | 66.81155 | 107.9561 | 1.07726 |
| 108 | male | 70~89 | 0.510927 | 0.625826 | 6.277915 | 39.91285 | 95.79037 | 1.27835 |
| 109 | male |  | 0.705221 | 0.782411 | 11.03013 | 42.0138 | 106.1791 | 1.12499 |
| 110 | male |  | 0.76172 | 0.857357 | 15.84005 | 38.6568 | 106.8168 | 1.17635 |
| 111 | male |  | 0.789299 | 0.984474 | 17.12715 | 49.37395 | 95.11444 | 1.25974 |
| 112 | male |  | 0.695389 | 0.998155 | 20.42735 | 50.79295 | 83.49352 | 1.45086 |
| 113 | male |  | 0.624962 | 0.779674 | 13.83745 | 38.0536 | 93.32911 | 1.3212 |
| 114 | male |  | 0.500947 | 0.599829 | 5.88658 | 34.81325 | 98.5917 | 1.19461 |
| 115 | male |  | 1.183958 | 1.332092 | 30.8092 | 63.8619 | 104.6475 | 1.13527 |
| 116 | male |  | 0.768163 | 1.014622 | 20.18155 | 50.638 | 93.46163 | 1.2819 |
| 117 | male |  | 0.691536 | 0.986735 | 16.2381 | 46.0063 | 83.95746 | 1.40289 |
| 118 | male |  | 0.857107 | 1.120586 | 20.80925 | 54.13985 | 89.29832 | 1.32388 |
| 119 | male |  | 0.354349 | 0.484801 | 3.03638 | 29.4585 | 88.20338 | 1.30792 |
| 120 | male |  | 0.393448 | 0.451029 | 1.090394 | 35.5432 | 106.1768 | 1.11595 |
| 121 | male |  | 0.826951 | 0.973472 | 19.62435 | 52.69555 | 102.3231 | 1.21549 |
| 122 | male |  | 0.786359 | 1.008756 | 19.8203 | 55.6474 | 92.4114 | 1.30581 |
| 123 | male |  | 0.810705 | 0.997786 | 13.5888 | 57.7691 | 95.71664 | 1.28164 |
| 124 | male |  | 0.702171 | 0.812388 | 7.885995 | 43.3005 | 100.8321 | 1.22977 |
| 125 | male |  | 0.842797 | 1.056021 | 19.1106 | 58.03765 | 92.6335 | 1.32104 |
| 126 | male |  | 1.166148 | 1.119211 | 13.5854 | 61.72775 | 123.4572 | 0.962167 |
| 127 | male |  | 0.931259 | 1.123329 | 16.63475 | 66.0855 | 98.09258 | 1.23916 |
| 128 | male |  | 1.150278 | 1.308362 | 21.37625 | 65.02715 | 105.7077 | 1.12932 |
| 129 | male |  | 1.261182 | 1.424123 | 28.4929 | 71.025 | 106.3527 | 1.12159 |
| 130 | male |  | 0.703116 | 0.902264 | 15.494 | 49.9211 | 92.60444 | 1.28807 |
| 131 | male |  | 1.113314 | 1.179834 | 22.9442 | 74.7842 | 112.4839 | 1.03698 |
| 132 | male |  | 1.252444 | 1.338164 | 25.60935 | 63.98715 | 111.259 | 1.06135 |
| 133 | male |  | 1.033778 | 1.202125 | 21.68015 | 66.97705 | 102.492 | 1.15623 |
| 134 | male |  | 1.130755 | 1.087535 | 16.03455 | 50.19795 | 123.2128 | 0.95534 |
| 135 | male |  | 1.007247 | 1.047289 | 20.4358 | 52.81405 | 114.2229 | 1.06476 |
| 136 | male |  | 1.03635 | 1.123529 | 18.4614 | 56.3084 | 109.0113 | 1.05703 |
| 137 | male |  | 0.97972 | 1.113126 | 14.5921 | 66.9102 | 104.2883 | 1.14561 |
| 138 | male |  | 1.156969 | 1.154324 | 21.77255 | 62.5604 | 119.2268 | 0.999046 |
| 139 | male |  | 1.092741 | 1.186601 | 17.94265 | 64.9567 | 108.7965 | 1.11204 |
| 140 | female | 20~29 | 0.893266 | 1.054516 | 17.2044 | 74.20335 | 100.972 | 1.19422 |
| 141 | female |  | 1.183336 | 1.139197 | 16.85775 | 69.7263 | 124.4144 | 0.969389 |
| 142 | female |  | 1.253823 | 1.229173 | 22.5291 | 76.08515 | 120.3723 | 1.00129 |
| 143 | female |  | 1.048993 | 1.033072 | 14.69095 | 68.14875 | 120.768 | 1.00177 |
| 144 | female |  | 0.990739 | 1.086448 | 18.3938 | 68.42845 | 107.9006 | 1.14727 |
| 145 | female |  | 1.238719 | 1.131132 | 19.3294 | 65.5591 | 131.2819 | 0.918304 |
| 146 | female |  | 1.039173 | 1.103487 | 20.67555 | 57.5179 | 110.9152 | 1.10458 |
| 147 | female |  | 1.267815 | 1.161788 | 24.0412 | 71.87695 | 131.1319 | 0.942248 |
| 148 | female |  | 1.18978 | 1.143896 | 16.9726 | 82.66675 | 124.7875 | 0.950682 |
| 149 | female |  | 1.032158 | 1.120374 | 14.2742 | 72.6576 | 109.4087 | 1.12058 |
| 150 | female |  | 0.937853 | 1.061083 | 20.6703 | 66.74415 | 105.437 | 1.17826 |
| 151 | female |  | 0.967912 | 1.109479 | 14.89265 | 73.16145 | 104.1644 | 1.17817 |
| 152 | female |  | 1.123015 | 1.199717 | 24.8939 | 72.5073 | 111.008 | 1.10275 |
| 153 | female |  | 1.135326 | 1.207605 | 23.58495 | 80.4955 | 112.9746 | 1.08708 |
| 154 | female |  | 0.885925 | 1.022262 | 17.38 | 66.27825 | 103.2815 | 1.19859 |
| 155 | female |  | 1.296017 | 1.241633 | 26.7743 | 74.15625 | 123.5467 | 0.972571 |
| 156 | female |  | 1.382624 | 1.362079 | 26.9174 | 68.90925 | 121.2632 | 1.00922 |
| 157 | female |  | 1.212086 | 1.248549 | 22.5374 | 72.21015 | 115.3624 | 1.06137 |
| 158 | female |  | 1.193411 | 1.252927 | 24.65505 | 68.35285 | 111.6606 | 1.11922 |
| 159 | female |  | 1.004689 | 1.107429 | 16.4061 | 58.44925 | 107.522 | 1.14183 |
| 160 | female | 30~39 | 1.042055 | 1.076531 | 18.61725 | 72.26615 | 116.001 | 1.03908 |
| 161 | female |  | 1.050164 | 1.197545 | 24.266 | 80.83035 | 104.9248 | 1.14986 |
| 162 | female |  | 1.084416 | 1.0701 | 14.4761 | 67.8632 | 120.5791 | 1.0033 |
| 163 | female |  | 1.242864 | 1.278072 | 20.14455 | 78.3184 | 115.0575 | 1.04733 |
| 164 | female |  | 1.058214 | 1.12285 | 16.6384 | 77.6727 | 112.2245 | 1.06822 |
| 165 | female |  | 0.886516 | 1.063261 | 19.3274 | 67.02305 | 100.0399 | 1.20458 |
| 166 | female |  | 1.072709 | 1.117816 | 24.1874 | 65.55415 | 113.7655 | 1.08213 |
| 167 | female |  | 1.236867 | 1.243524 | 13.5082 | 74.0171 | 118.561 | 1.03219 |
| 168 | female |  | 1.203976 | 1.140787 | 23.45035 | 76.03225 | 126.2765 | 0.948438 |
| 169 | female |  | 0.99105 | 1.139352 | 21.78345 | 60.8842 | 103.028 | 1.17483 |
| 170 | female |  | 1.242405 | 1.163814 | 16.86985 | 62.33005 | 126.8868 | 0.953313 |
| 171 | female |  | 1.307672 | 1.287968 | 20.94315 | 74.3384 | 121.5286 | 0.991557 |
| 172 | female |  | 1.17413 | 1.117282 | 24.3446 | 67.90475 | 128.462 | 0.932701 |
| 173 | female |  | 1.156467 | 1.167366 | 20.42385 | 65.1602 | 118.9701 | 1.01356 |
| 174 | female |  | 1.311084 | 1.218681 | 19.4204 | 59.8615 | 126.7949 | 0.935354 |
| 175 | female |  | 1.08738 | 1.164519 | 21.4011 | 76.5983 | 110.6189 | 1.10046 |
| 176 | female |  | 0.950579 | 1.058998 | 19.8061 | 68.36325 | 106.9011 | 1.1287 |
| 177 | female |  | 1.18975 | 1.184362 | 19.2218 | 63.71305 | 120.2025 | 0.986884 |
| 178 | female |  | 1.24119 | 1.243491 | 24.74215 | 70.9453 | 119.4932 | 1.00887 |
| 179 | female |  | 1.137387 | 1.150409 | 17.5804 | 71.63705 | 117.6426 | 1.04092 |
| 180 | female | 40~49 | 0.920304 | 0.969882 | 18.48975 | 64.90195 | 113.0093 | 1.08634 |
| 181 | female |  | 0.974048 | 1.104016 | 12.4846 | 71.3565 | 104.9067 | 1.15037 |
| 182 | female |  | 0.986128 | 1.030607 | 16.53085 | 66.52305 | 114.0664 | 1.04017 |
| 183 | female |  | 0.926614 | 1.071272 | 24.03325 | 57.5662 | 103.1229 | 1.16239 |
| 184 | female |  | 0.920864 | 1.013891 | 14.26275 | 60.9039 | 109.0145 | 1.07866 |
| 185 | female |  | 0.869444 | 1.039043 | 12.3411 | 65.32475 | 99.74321 | 1.2011 |
| 186 | female |  | 1.215715 | 1.300168 | 26.6459 | 87.5475 | 111.9017 | 1.07367 |
| 187 | female |  | 1.124559 | 1.192092 | 18.05415 | 65.40345 | 112.1329 | 1.07153 |
| 188 | female |  | 1.127761 | 1.163111 | 18.7092 | 86.85475 | 116.3121 | 1.03091 |
| 189 | female |  | 1.002722 | 1.015846 | 15.96385 | 67.3637 | 117.6794 | 1.01451 |
| 190 | female |  | 1.199174 | 1.218106 | 21.9963 | 65.6354 | 117.1549 | 1.02232 |
| 191 | female |  | 0.927134 | 1.028993 | 21.70995 | 67.7148 | 107.4088 | 1.10465 |
| 192 | female |  | 1.02022 | 1.066924 | 15.5029 | 66.12345 | 113.6693 | 1.07168 |
| 193 | female |  | 1.056056 | 1.113717 | 16.02605 | 74.8655 | 113.7147 | 1.04813 |
| 194 | female |  | 1.153101 | 1.120065 | 19.5172 | 69.58105 | 123.5273 | 0.953755 |
| 195 | female |  | 1.077523 | 1.095636 | 17.02825 | 64.36615 | 118.1334 | 1.0165 |
| 196 | female |  | 1.243722 | 1.27699 | 21.49035 | 66.06565 | 116.6258 | 1.00088 |
| 197 | female |  | 1.08537 | 1.11281 | 17.26245 | 62.90505 | 115.6116 | 1.02653 |
| 198 | female |  | 1.009997 | 1.152338 | 18.8809 | 78.3669 | 104.2392 | 1.14304 |
| 199 | female |  | 1.188763 | 1.182334 | 19.94625 | 66.31335 | 119.9071 | 1.00845 |
| 200 | female |  | 1.147015 | 1.250821 | 27.3797 | 76.4911 | 109.28 | 1.08949 |
| 201 | female | 50~59 | 1.205925 | 1.180539 | 20.7429 | 66.7076 | 120.2726 | 1.00648 |
| 202 | female |  | 1.08868 | 1.084025 | 17.7135 | 65.1885 | 118.8016 | 1.011 |
| 203 | female |  | 1.300871 | 1.307855 | 24.4217 | 72.7092 | 118.8915 | 0.998236 |
| 204 | female |  | 1.248525 | 1.337537 | 24.2026 | 72.28495 | 111.9627 | 1.08371 |
| 205 | female |  | 1.408004 | 1.369602 | 19.23215 | 81.94075 | 122.3216 | 0.969862 |
| 206 | female |  | 1.032441 | 1.122938 | 22.79145 | 68.214 | 109.7383 | 1.08093 |
| 207 | female |  | 1.197748 | 1.214972 | 9.962135 | 75.6808 | 117.9965 | 1.04009 |
| 208 | female |  | 1.132825 | 1.133142 | 16.55735 | 64.4928 | 119.1188 | 1.02203 |
| 209 | female |  | 0.983025 | 1.070932 | 18.1006 | 69.4376 | 109.004 | 1.10295 |
| 210 | female |  | 1.157898 | 1.197481 | 12.8164 | 81.1366 | 116.0527 | 1.03287 |
| 211 | female |  | 1.166712 | 1.168022 | 19.50275 | 65.22535 | 118.5024 | 1.02433 |
| 212 | female |  | 1.26937 | 1.217836 | 19.5553 | 74.4485 | 124.7298 | 0.981156 |
| 213 | female |  | 1.129968 | 1.15169 | 22.864 | 67.25915 | 117.1604 | 0.992558 |
| 214 | female |  | 1.086528 | 1.028064 | 12.15275 | 76.10025 | 125.9732 | 0.956542 |
| 215 | female |  | 1.212248 | 1.172379 | 25.6068 | 64.2311 | 124.1032 | 0.972347 |
| 216 | female |  | 1.280363 | 1.192995 | 21.13775 | 73.1842 | 128.7187 | 0.906264 |
| 217 | female |  | 1.164726 | 1.193078 | 18.8142 | 73.70045 | 117.4638 | 1.05184 |
| 218 | female |  | 1.170886 | 1.132801 | 17.1417 | 65.7412 | 123.9584 | 0.98125 |
| 219 | female |  | 1.278949 | 1.175043 | 18.57235 | 72.0005 | 130.275 | 0.907789 |
| 220 | female |  | 1.204859 | 1.218901 | 19.0451 | 74.8984 | 117.8006 | 1.00992 |
| 221 | female |  | 1.198643 | 1.20023 | 15.97075 | 68.422 | 119.1753 | 0.998331 |
| 222 | female |  | 1.21806 | 1.209433 | 17.85165 | 76.83405 | 120.2785 | 1.01725 |
| 223 | female |  | 1.189982 | 1.190533 | 15.7835 | 71.711 | 119.0614 | 1.01228 |
| 224 | female |  | 1.137059 | 1.166398 | 16.2783 | 80.7414 | 116.1757 | 1.03737 |
| 225 | female | 60~69 | 1.12359 | 1.079791 | 15.42825 | 67.5514 | 123.9589 | 0.969476 |
| 226 | female |  | 1.093125 | 1.167569 | 22.44815 | 65.7551 | 111.1588 | 1.09472 |
| 227 | female |  | 1.224653 | 1.212969 | 17.06095 | 78.25205 | 121.7798 | 0.996345 |
| 228 | female |  | 1.134017 | 1.233893 | 22.61765 | 73.6504 | 109.9241 | 1.05429 |
| 229 | female |  | 0.949099 | 1.068506 | 14.68545 | 59.7764 | 105.0729 | 1.13779 |
| 230 | female |  | 1.094991 | 1.087681 | 17.82655 | 72.8521 | 119.9529 | 0.98556 |
| 231 | female |  | 0.977388 | 1.015 | 12.74405 | 63.8476 | 113.0533 | 1.08585 |
| 232 | female |  | 1.006397 | 1.104405 | 15.2912 | 74.4228 | 108.9202 | 1.0925 |
| 233 | female |  | 1.131482 | 1.168648 | 19.9024 | 63.22005 | 115.2117 | 1.02282 |
| 234 | female |  | 0.939225 | 0.941038 | 13.2234 | 51.1817 | 117.9292 | 1.0222 |
| 235 | female |  | 1.256554 | 1.184751 | 16.9225 | 71.622 | 126.1127 | 0.961597 |
| 236 | female |  | 1.13984 | 1.08074 | 20.60915 | 71.56115 | 125.777 | 0.959021 |
| 237 | female |  | 1.099107 | 1.137697 | 12.98445 | 70.9932 | 114.4261 | 1.06331 |
| 238 | female |  | 1.306227 | 1.207865 | 23.67385 | 76.83605 | 129.5044 | 0.922843 |
| 239 | female |  | 1.102579 | 1.118747 | 23.6873 | 72.68165 | 116.9526 | 1.04708 |
| 240 | female |  | 1.330596 | 1.271251 | 27.0955 | 71.86325 | 123.9439 | 0.941592 |
| 241 | female |  | 1.035741 | 1.02158 | 16.5155 | 53.4499 | 120.5532 | 0.983858 |
| 242 | female |  | 1.156218 | 1.142076 | 21.56125 | 71.72725 | 119.9931 | 1.0136 |
| 243 | female |  | 1.173342 | 1.167553 | 15.7081 | 69.24425 | 119.2139 | 0.978655 |
| 244 | female |  | 1.425016 | 1.234535 | 23.7417 | 65.92945 | 136.4548 | 0.89682 |
| 245 | female |  | 1.130853 | 1.12411 | 20.41685 | 61.53105 | 120.4897 | 1.00012 |
| 246 | female |  | 1.081657 | 1.19031 | 25.3196 | 62.9283 | 109.0175 | 1.07734 |
| 247 | female |  | 1.100194 | 1.027544 | 21.3696 | 62.9684 | 127.1118 | 0.945349 |
| 248 | female |  | 1.139826 | 1.224162 | 22.10155 | 67.21275 | 111.6832 | 1.09292 |
| 249 | female |  | 1.220087 | 1.108055 | 19.84685 | 66.71525 | 130.005 | 0.921172 |
| 250 | female |  | 1.224006 | 1.183884 | 19.0075 | 64.0197 | 123.3558 | 0.994988 |
| 251 | female |  | 1.393683 | 1.450464 | 28.5182 | 83.7321 | 115.2333 | 1.02274 |
| 252 | female |  | 1.059519 | 1.142676 | 13.68395 | 58.314 | 109.6985 | 1.09851 |
| 253 | female | 70~89 | 0.60023 | 0.715046 | 8.419345 | 43.319 | 98.56026 | 1.23153 |
| 254 | female |  | 0.732683 | 0.744334 | 6.77076 | 47.41335 | 116.3598 | 1.00953 |
| 255 | female |  | 0.739337 | 0.822835 | 13.5372 | 48.78365 | 106.538 | 1.13926 |
| 256 | female |  | 0.693134 | 0.629705 | 6.68883 | 39.2891 | 130.0865 | 0.92741 |
| 257 | female |  | 0.616893 | 0.835925 | 12.7944 | 49.1754 | 87.72853 | 1.3311 |
| 258 | female |  | 0.820302 | 0.990506 | 19.0111 | 48.477 | 98.06315 | 1.19059 |
| 259 | female |  | 0.50445 | 0.61722 | 6.979035 | 42.74955 | 95.80172 | 1.21925 |
| 260 | female |  | 0.715369 | 0.784092 | 13.98445 | 44.6049 | 108.8907 | 1.08931 |
| 261 | female |  | 0.370998 | 0.554437 | 8.248805 | 40.0097 | 79.39283 | 1.5482 |
| 262 | female |  | 0.877514 | 1.009238 | 18.16805 | 63.2557 | 101.8395 | 1.12773 |
| 263 | female |  | 0.888546 | 0.988665 | 16.4273 | 52.98965 | 106.5299 | 1.13882 |
| 264 | female |  | 0.453863 | 0.565215 | 4.533065 | 33.634 | 95.64132 | 1.2423 |
| 265 | female |  | 0.897457 | 0.978531 | 12.0928 | 59.0259 | 106.9521 | 1.10214 |
| 266 | female |  | 0.725452 | 0.778682 | 10.6671 | 51.01485 | 110.5598 | 1.08426 |
| 267 | female |  | 1.02849 | 1.066621 | 14.5401 | 62.05745 | 114.5345 | 1.01554 |
| 268 | female |  | 0.985354 | 1.030369 | 14.14705 | 66.4997 | 114.6115 | 1.02483 |
| 269 | female |  | 0.503983 | 0.554652 | 5.844 | 34.93285 | 106.8772 | 1.12257 |
| 270 | female |  | 1.03737 | 1.04988 | 12.78115 | 56.90765 | 115.3803 | 1.03173 |
| 271 | female |  | 0.705454 | 0.857319 | 11.8284 | 46.39325 | 96.51115 | 1.21442 |
| 272 | female |  | 0.493656 | 0.510289 | 4.109625 | 30.02195 | 114.1544 | 1.05034 |
| 273 | female |  | 0.889515 | 0.92386 | 15.0475 | 55.58695 | 113.4035 | 1.05729 |
| 274 | female |  | 0.659506 | 0.881364 | 15.30595 | 57.93015 | 89.81864 | 1.33337 |
| 275 | female |  | 0.965089 | 1.030043 | 12.3787 | 59.39245 | 111.7146 | 1.10233 |
| 276 | female |  | 0.953659 | 1.09573 | 22.6926 | 61.5152 | 104.6445 | 1.17118 |
| 277 | female |  | 1.076612 | 1.124042 | 11.988 | 66.88605 | 113.6359 | 1.07051 |
| 278 | female |  | 0.815871 | 0.938401 | 17.95045 | 54.75345 | 102.6746 | 1.15506 |
| 279 | female |  | 1.104662 | 1.128791 | 21.4619 | 72.65515 | 116.9983 | 1.02455 |
| 280 | female |  | 0.865548 | 0.929819 | 15.45555 | 64.1981 | 109.1366 | 1.09314 |
| 281 | female |  | 0.836353 | 0.93641 | 11.90445 | 66.4993 | 105.578 | 1.13145 |
| 282 | female |  | 0.841758 | 0.941574 | 11.50285 | 57.07135 | 107.1572 | 1.10004 |
| 283 | female |  | 0.906936 | 1.02195 | 11.91385 | 73.2208 | 105.1047 | 1.14431 |
| 284 | female |  | 0.934586 | 1.00826 | 20.15665 | 57.36035 | 110.5672 | 1.05549 |
| 285 | female |  | 1.184142 | 1.175002 | 20.0051 | 71.30555 | 120.4567 | 1.02249 |
| 286 | female |  | 1.062339 | 1.097538 | 10.98021 | 63.7264 | 114.9529 | 1.06868 |
| 287 | female |  | 1.133058 | 1.117877 | 19.6801 | 74.1376 | 120.7173 | 0.966496 |
| 288 | female |  | 0.924976 | 1.04451 | 16.0744 | 67.20905 | 105.7875 | 1.10368 |
| 289 | female |  | 1.126472 | 1.150113 | 19.2701 | 67.1413 | 115.5894 | 1.02972 |
| 290 | female |  | 1.158161 | 1.191565 | 21.0018 | 67.212 | 115.9739 | 1.0394 |
| 291 | female |  | 1.240869 | 1.122922 | 15.4451 | 67.4457 | 130.353 | 0.913383 |
| 292 | female |  | 0.822135 | 1.045229 | 17.41495 | 71.58405 | 93.6192 | 1.28154 |
